# An iterative tomosynthesis reconstruction using total variation combined with non-local means filtering

**DOI:** 10.1186/1475-925X-13-65

**Published:** 2014-05-27

**Authors:** Metin Ertas, Isa Yildirim, Mustafa Kamasak, Aydin Akan

**Affiliations:** 1 Electrical and Electronics Engineering Department, Istanbul University, Avcilar, 34320 Istanbul, Turkey; 2 Electrical and Electronics Engineering Department, Istanbul Technical University, Maslak, 34469 Istanbul, Turkey; 3 College of Engineering Department, University of Illinois at Chicago, Chicago, IL 60607, USA; 4 Computer Engineering Department, Istanbul Technical University, Maslak, 34469 Istanbul, Turkey

**Keywords:** Non local means, ART, Tomosynthesis, Total variation, Compressed sensing

## Abstract

**Background:**

After the release of compressed sensing (CS) theory, reconstruction algorithms from sparse and incomplete data have shown great improvements in diminishing artifacts of missing data. Following this progress, both local and non-local regularization induced iterative reconstructions have been actively used in limited view angle imaging problems.

**Methods:**

In this study, a 3D iterative image reconstruction method (ART + TV)_NLM_ was introduced by combining local total variation (TV) with non-local means (NLM) filter. In the first step, TV minimization was applied to the image obtained by algebraic reconstruction technique (ART) for background noise removal with preserving edges. In the second step, NLM is used in order to suppress the out of focus slice blur which is the most existent image artifact in tomosynthesis imaging. NLM exploits the similar structures to increase the smoothness in the image reconstructed by ART + TV.

**Results:**

A tomosynthesis system and a 3D phantom were designed to perform simulations to show the superior performance of our proposed (ART + TV)_NLM_ over ART and widely used ART + TV methods. Visual inspections show a significant improvement in image quality compared to ART and ART + TV.

**Conclusions:**

RMSE, Structure SIMilarity (SSIM) value and SNR of a specific layer of interest (LOI) showed that by proper selection of NLM parameters, significant improvements can be achieved in terms of convergence rate and image quality.

## Background

Dose reduction in imaging with ionizing radiation has been an increasing concern lately due to its potential risk of causing radiation related cancers. For tomographic imaging; lowering the dose [1], taking fewer projections [2] and limiting the scan angle [2,3] during the acquisition have been introduced as solutions for dose reduction in both experimental and clinical studies. However all of these techniques cause severe artifacts in the reconstructed images leading to less reliable clinical images. To be able to deal with the radiation dose and image quality trade-off advanced reconstruction techniques need to be developed. Analytical algorithms such as Fourier transform (FT) and filtered back projection (FBP) are not sufficient enough to reconstruct an artifact-free image due to high amount of missing data. Iterative methods have been proposed to overcome this problem. Numerous iterative algorithms have been applied to tomographic imaging such as; expectation-maximization (EM) [2], projection onto convex sets (POCS) [3], algebraic reconstruction technique (ART) [2,4,5], simultaneous algebraic reconstruction technique SART [6]. However iterative reconstruction techniques themselves are also not enough to reconstruct artifact-free images and therefore further improvements are required to obtain improved results. Compressed sensing (CS) approach which demonstrated the feasibility to reconstruct signals using highly incomplete measurement data via optimization methods has been introduced [7,8]. Among these optimization methods, total variation (TV) [9] minimization has been widely used in CT, MRI, tomosynthesis modalities under the light of CS framework [2-5].

TV considers how intensities change in the image. In most medical images, within an organ or tissue, the intensity does not change dramatically due to uniformity and rapid variation occurs at boundaries of organs and tissues. Thus minimizing the TV of an image significantly preserves edges and creates a smoother image. To improve the performance of TV for specific problems several approaches and constraints have been included. PICCS which was introduced by adding a prior image as constraint to the optimization problem showed superior results over CS and FBP methods [4]. TV was replaced with anisotropic TV (ATV) for images which contain different resolutions along each axis, thus by weighting the terms in TV with respect to each axis resulted in better images in terms of SNR when it is compared to SART and SART + TV [6]. TV minimization can be considered as minimizing the variation between neighboring pixels and therefore can be named as local total variation; however in recently proposed nonlocal total variation (NLTV), the regularization is applied to pixels in the entire image instead of applying to neighboring pixels only and it has been proven to have better results for research and clinical problems over local TV [9-11]. In addition to local and nonlocal regularization methods for denoising, nonlocal means (NLM) filter has been used in image denoising [11,12]. Most denoising methods remove fine structures, textures and details in the image; however it has been shown that NLM gives far better results than other spatial image denoising algorithms in preserving of fine structures and objects. In order to enhance the historical printed document which contains lots of redundancy, NLM was combined with TV and the new method performed better than both NLM and TV individually [13]. NLM was also integrated to iterative methods in sparse CT reconstruction by applying NLM after each POCS iteration and significant edge preservation were shown [14]. It has also been shown that using NLM with statistical iterative reconstructions (SIR) achieved better results than SIR and FBP in terms of resolution and noise reduction [15].

In this study, a widely used sparse image reconstruction algorithm ART + TV was modified with NLM filter to reduce the out-of-focus slice blur in tomosynthesis system. Tomosynthesis is an imaging modality which produces 3D images of breast by using 2D projections taken from a limited view angle. The angular view varies from 15° to 50° in some commercial tomosynthesis modalities [16]. Thus, out-of-focus slice blur is the most dominant artifact in tomosynthesis system wherein fine structures in a specific slice of interest are blurred or more practically overlapped by other structures in upper and lower slices. Our study consists of introducing a new way to reduce the out-of-focus blur in tomosynthesis by applying TV and NLM sequentially in an iterative manner. In the first step, 3D TV minimization was applied to the image reconstructed by ART to reduce the background noise and sharpen the edges. In the second step, image reconstructed with ART + TV was filtered by NLM to make small details and fine objects more visible. NLM is applied to each slice independently in 2D form to fully cover the 3D image. A 3D phantom was designed for performance comparison of newly introduced (ART + TV)_NLM_ method with other widely used iterative tomosynthesis reconstruction techniques ART and ART + TV. Both qualitative and quantitative comparisons were performed to show (ART + TV)_NLM_ provides superior results than ART and ART + TV.

The rest of the paper is organized as follows. The following section introduces ART, TV minimization, NLM filter and (ART + TV)_NLM_ method. System design, quantitative and qualitative results are shown in Section ‘‘Numerical Experiments’’. Section ‘‘Discussion and Conclusion’’ concludes the paper.

## Methods

### Algebraic Reconstruction Technique (ART)

ART is one of the simplest and most commonly used iterative reconstruction techniques [17]. In ART, an image is estimated by minimizing:

(1) $$\hat{X}=\underset{X}{\arg\min}\left({∥Y-\mathrm{AX}∥}^{2}\right)$$

where *Y* is the measured data, $\hat{X}$ is the image to be estimated and *A* is the system matrix which can also be considered as the weighting matrix. It basically uses sequential sets of projection data to estimate the image *X* from an initial estimation. An image is updated by using the following formulation:

(2) $$X_{j}^{\left(k+1\right)}=X_{j}^{\left(k\right)}+\frac{Y_{i}-\sum_{k=1}^{N}A_{\mathrm{ik}}X_{k}^{\left(k\right)}}{\sum_{k=1}^{N}A_{\mathrm{ik}}^{2}}A_{\mathrm{ij}},\;\begin{array}{c} i=1,2,\ldots ,M\\ j=1,2,\ldots ,N \end{array}$$

where $X_{j}^{\left(k\right)}$ and $X_{j}^{\left(k+1\right)}$ show the previous (or initial for the first iteration) and current images respectively. *Y*_*i*_ is the projection data corresponding to the *i*^*th*^ ray integral. *i* and *j* are ray and voxel indexes respectively. *M* is the total number of rays and *N* shows the number of voxels. *A*_*ij*_ is the weighting parameter which gives the influence of *j*^*th*^ voxel on the *i*^*th*^ ray line integral. Weighting parameter is calculated by using the Siddon’s algorithm [18]. This algorithm calculates the contribution of voxels to the corresponding radiological path of a ray. Thus this data is used as system matrix in (1).

In (2), an image $X_{j}^{\left(k+1\right)}$ is updated from $X_{j}^{\left(k\right)}$ by adding a calculated error value. The error value is the normalized difference between the measured projection data *Y*_*i*_ and the calculated projection data $\sum_{k=1}^{N}A_{\mathrm{ik}}X_{k}^{\left(k\right)}$. To complete a single iteration, the update process is repeated for all projections. The reconstruction algorithm continues until a convergence criterion is satisfied.

### Total Variation (TV) minimization

CS opened a new era in reconstruction problems. By CS, it was mathematically proven that an image or signal can be recovered from a highly undersampled data. This theory originated a new word “sparsity” for digital information processing. The theory says that an image can be accurately reconstructed from undersampled observations assuming the image is sparse. However not all images are sparse enough due to their natural structure. Thus a sparsifying transform might be needed to create a sparse image. In most medical images, the intensity variations happen at boundaries of structures. Thus taking gradient of an image can be used as a sparsifying transform. Summation of absolute values of discrete gradient of an image is represented as TV of an image. For a 3D image *TV*(*X*) is formulated as:

(3) $$\mathrm{TV}\left(X\right)=\sum_{k}^{K}\sum_{j}^{L}\sum_{i}^{I}\sqrt{{\left(X_{i,j,k}-X_{i-1,j,k}\right)}^{2}+{\left(X_{i,j,k}-X_{i,j-1,k}\right)}^{2}+{\left(X_{i,j,k}-X_{i,j,k-1}\right)}^{2}}$$

where *i*, *j*, *k* represent the coordinates and *K*, *L*, *I* show the number of voxels in each direction. *X*_*i*,*j*,*k*_ shows the intensity value of *X* at voxel (*i*, *j*, *k*). Adding TV of an image as a regularization term to (1), the minimization problem is modified to:

(4) $$\hat{X}=\min_{X}\;\left[{∥Y-\mathrm{AX}∥}_{2}+\lambda {∥\mathrm{\psi X}∥}_{1}\right]\;$$

where *λ* is the regularization parameter controlling impact of the TV term in the estimation. *ψ* represents the discrete gradient transform which is used as a sparsifying transform.

### Non Local Means (NLM) filter

The non-local filtering method was first applied by restoring a pixel by using similar neighboring pixels [19]. This idea was extended to a more generalized form by using a patch centered at each pixel rather than using the pixel itself. The NLM method bases on averaging the neighboring patches however this process is highly depended on the similarities of patches between neighboring pixels. Thus considering the entire image the NLM process can be extremely time consuming. Search windows are used to reduce the computational time as the similarity between remote patches is redundant for denoising purpose. The denoising process is repeated pixel by pixel for the entire image and formulated as:

(5) $$\mathrm{NLM}\left(X_{i}\right)=\sum_{X_{j}\in \mathrm{SW}}w\left(X_{i},X_{j}\right).\mu \left(X_{j}\right)$$

where *X*_*i*_ and *X*_*j*_ are the intensity values of image *X* at pixels *i* and *j. X*_*j*_ is limited to a search window (*SW*) which bounds the neighboring pixels remoteness. *μ* denotes the intensity value of specific pixel at image *X. w*(*X*_*i*_, *X*_*j*_) represents the weighting function between pixels *i* and *j*. The weighting function shows how much the difference between pixels *i* and *j* is penalized and it is calculated by using the following formulation:

(6) $$w\left(X_{i},X_{j}\right)=\frac{1}{C\left(X\right)}\sum_{\delta \in P}e^{-\frac{G_{a}{\left|\mu \left(X_{i}+\delta \right)-\mu \left(X_{j}+\delta \right)\right|}^{2}}{h^{2}}}$$

where *G*_*a*_ is the Gaussian kernel and *h* is the filtering parameter which controls the power of the filter and it is usually related to the level of noise in the image. *δ* represents the patch (*P*) centered at pixels *i* and *j. C*(*X*) is the normalization factor and calculated by summing up all weighting function values between the center pixel *i* and all other reference pixels *j* within the reference search window.

(7) $$C\left(X\right)=\sum_{X_{j}\in \mathrm{SW}}w\left(X_{i},X_{j}\right)$$

**Figure 1:**
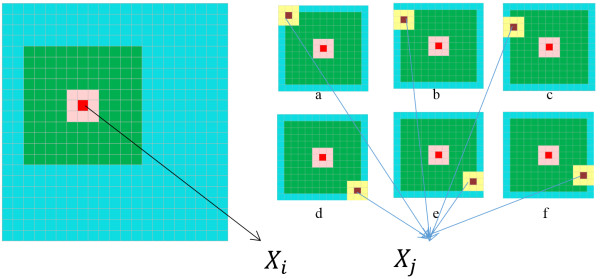
**Non-local Means filtering visualization.***Left side*: Original image, *right side* from ***a*** to ***f***: NLM filtering steps. (Please see the electronic version for color notations).

Figure 1 shows the visualization of how NLM is implemented for an image. The implementation of NLM starts by setting a search window centered at “red” pixel *i*. Search window can be considered as the reference image for that pixel. A small size of patch centering at the coordinate *i* is applied. To apply NLM to *X*_*i*_, a small size of patch centering at the coordinate *j* moves throughout the search window while patch centering at *i* remains unchanged. The weighting coefficients in (6) are calculated for all pixels in the reference image and *C*(*X*), the normalization factor in (7) is calculated by summing up all weighting factors for the search window. The final step is the recovery of *X*_*i*_ by using (5). This procedure is repeated for all pixels to denoise the image.

The size of patch and search window have a strong effect on denoising process. An increase in the size of the patch makes it unlikely to find similar patches within the search window. The similar behavior is observed with the change in size of the search window. A larger search window allows more similar patches to be found and leads a smoother image. However if the search window size is chosen too wide, the filter tends to oversmooth and results in loss of structures and fine details in the image. Increasing the search window size too much also increases the computational time. Optimization of the filter requires the knowledge of the level of noise in the image and physical resolution of the image. There have been many studies focusing on optimizing the parameters in NLM [20-22].

### Combining Total Variation (TV) and Non Local Means (NLM) filter

In this study, a widely used iterative reconstruction algorithm ART + TV for tomosynthesis was combined with NLM to reduce the out-of-focus slice blur which is the most dominant artifact in tomosynthesis imaging since the number of projection is limited to 11 with a scanning view of 50°. The blur reduces the visibility of small objects and fine details and creates oscillations at edges. Moreover using iterative methods for solving the limited view angle problems also creates a high level background noise due to high amount of missing data. The out-of-focus slice blur occurs at the superposition of objects wherein small details can be obscured by other dense objects. By using TV minimization the edges will be preserved while removing the background noise out to some extent. But the level of smoothing parameter in TV can cause losing small objects and fine details in the image. As a result, an improved method was needed to reduce the blur and background noise more effectively. This problem was aimed to be solved by integrating NLM to the TV regularized image in a sequential way. This algorithm can be divided into three major consecutive steps:

(8) $$\overset{*}{X}=\min_{X}\;\left[{∥Y-\mathrm{AX}∥}_{2}+\lambda {∥\mathrm{\psi X}∥}_{1}+{∥X∥}_{\mathrm{NLM}}\right],$$

**Figure 2:**
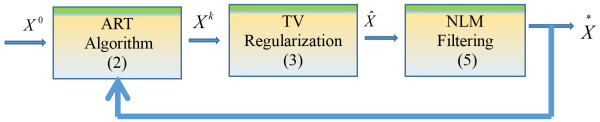
**Flow chart of (ART + TV)**_**NLM **_**algorithm.**

1. ART reconstruction: This step is done by applying (2) while satisfying the consistency condition in (1).


2. TV minimization: By solving the minimization problem in (4) ART + TV reconstruction is completed. In order to solve (4), the classical steepest descent algorithm is used. In this step, the edges will be preserved while removing the background noise out to some extent. It performs better results than ART, but it should be noted that small objects can also be diminished by TV.

3. NLM filtering: In tomosynthesis imaging, small structures with low absorption coefficients are blurred by other structures with higher absorption coefficients in upper slices. Thus, small objects are unlikely to be detected in a blurry image due to this overlapping problem. To make small objects more visible with less blur and noise, NLM filter is applied to the reconstructed image by ART + TV. In (4) $\hat{X}$ represents the image reconstructed by ART + TV. By filtering $\hat{X}$ with NLM by using (5), minimization problem becomes:

The main goal of the algorithm is to introduce a 3D iterative reconstruction technique which uses ART for image acquisition, TV for regularization and NLM for filtering to reduce the out-of-focus slice blur in the image. Both ART and TV are applied in 3D form. However NLM algorithm is applied layer by layer in 2D form to fully cover the 3D image. Figure 2 shows the flow chart of (ART + TV)_NLM_ algorithm.

## Numerical experiments

### Experiment setup

In order to perform the simulations, we considered a tomosynthesis system with rotating geometry. Both X-ray source and detector rotate along the same direction with step and shoot data acquisition. The source to the center of the object and detector are 300 and 355 pixels, respectively. The characteristics of tomosynthesis systems show differences for image acquisition such as: number of projections, angular range, reconstruction methods and so on [16]. Among these parameters, angular range and number of projections have much stronger influence on the reconstructed image quality. In our study, the angular range was considered 50° covering a scan view from −25° to 25° with an increment of 5° in each projection. By increasing the projection angle 5°, projection number is limited to 11 projections. To have a better consistency with our previous study and for convenience, the phantom used in [5] was considered as the phantom of this study. The phantom consists of 10 layers where small objects with low X-ray absorption coefficients were obscured by objects with higher X-ray absorption coefficients to show the tissue overlapping problem in breast imaging.

An experience-based fixed regularization parameter *λ* was set to 0.8 for ART + TV and (ART + TV)_NLM_ methods in our experiments. Parameter selection for NLM is very important as it shows significant effect on the filtered image. There have been studies addressing adaptive selection and optimization of parameters in NLM [20-22]. For this study, parameters required for NLM filtering was set constant to 11 and 15 for patch size *P* and search window *SW* respectively while the filtering parameter *h* was chosen 0.8 for the smoothing level.

The main limitation of 3D tomosynthesis is not the system noise but noise caused by the out-of-slice blur. Thus all simulations were carried out for noise-free case. The number of iteration was limited to 10 for all simulations as the radiologists can reliably comment on the clinical results obtained after 8–10 iterations with no further improvement in image quality after 10 iterations [1].

All simulations were performed in MATLAB® software on a system configuration of Intel (RM) Core(TM) i7-2630 QM CPU @ 2.00 GHz CPU, 6 GB Memory, Windows 7 64 Bits operating system. The performance of (ART + TV)_NLM_ was compared with ART and ART + TV in terms of SNR, SSIM and RMSE values of a specific layer of interest. Performance of three reconstruction methods was compared both visually and quantitatively.

### Visual comparison

**Figure 3:**
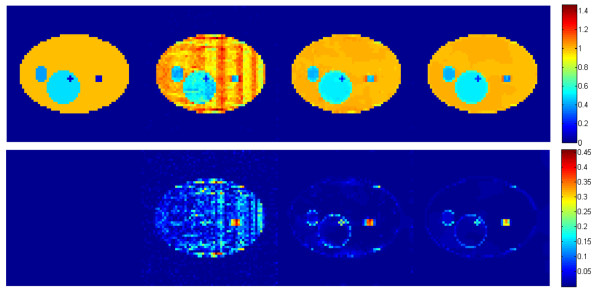
**Reconstruction results of the 3rd layer (LOI).***From left to right column*: original layer, images reconstructed by ART, ART + TV and (ART + TV)_NLM_, respectively. The first row shows the reconstruction results. The second row shows the absolute differences of reconstructed images relative to the original image.

**Figure 4:**
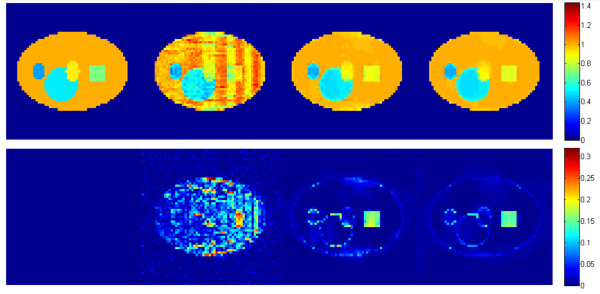
**Reconstruction results of the 7th layer.***From left to right column*: original layer, images reconstructed by ART, ART + TV and (ART + TV)_NLM_, respectively. The first row shows the reconstruction results. The second row shows the absolute differences of reconstructed images relative to the original image.

For the sake of consistency, the system parameters were considered the same for three reconstruction algorithms. In order to visualize the reconstruction results on 3D image, the reconstructed images of a specific layer of interest (3rd layer) and 7th layer were presented in Figures 3 and 4 respectively. In each figure, the first row shows the reconstruction results while the second row shows the absolute difference between the reconstructed image and the original image. To increase the visual awareness for the comparison, the original phantom was included in the first column in both figures. The second, third and fourth columns show images reconstructed by ART, ART + TV, (ART + TV)_NLM_ respectively.

**Figure 5:**
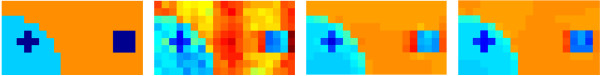
**3X zoom of a region in the 3rd layer.***From left to right:* Original image, reconstructed images by ART, ART+TV, (ART + TV)NLM.

The goal of this work is to reduce the most dominant artifact in tomosynthesis system, the out-of-focus slice blur. In Figures 3 and 4, it can be clearly seen that the TV inclusion significantly reduces the background noise and preserves edges. However in Figure 3 the out-of-focus slice blur is still existent around the small square object at the right side of the image reconstructed by ART + TV. As shown in Figure 3, the intensities of small star and square objects become closer to their original values by integrating NLM to ART + TV. Moreover, the reconstructed image becomes smoother than the image reconstructed by ART + TV and the blur is also reduced while preserving edges. The second rows in Figures 3 and 4 show that (ART + TV)_NLM_ reconstructed an image with the least background noise. Figure 5 shows the 3X zoomed images of the small objects in LOI to present the reduction of out-of-focus-slice blur. The results show that (ART + TV)_NLM_ not only reduces the blurring artifact but also provides the closest intensity value to the original object intensities. However, human observations are always prone to bias. To increase the stability of visual observations several quantitative analysis have been performed.

### Quantitative results comparison

**Figure 6:**
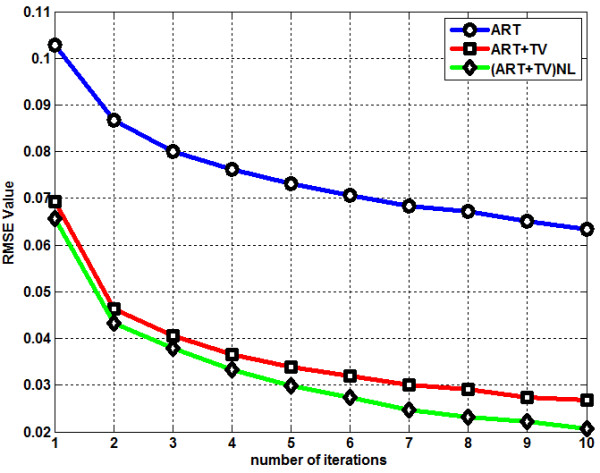
RMSE graph of the reconstruction methods.

**Figure 7:**
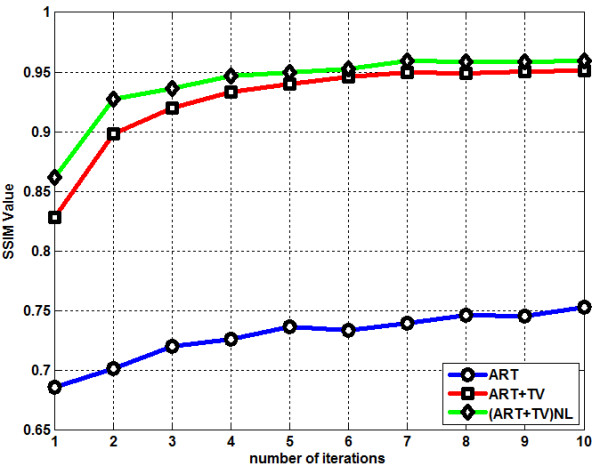
SSIM graph of the reconstruction methods.

The quantitative comparison of ART, ART + TV and (ART + TV)_NLM_ reconstruction methods is assessed using RMSE of a specific LOI by using the following formulation for a 2D image:

(9) $$\mathrm{RMSE}=\sqrt{\sum_{j,k}^{J,K}{\left(X_{\mathrm{jk}}-X_{{}_{\mathrm{jk}}}^{\mathrm{rec}}\right)}^{2}/N}$$

where *N* shows the number of pixels in the image. *X* and *X*^*rec*^ represent the original and the reconstructed images respectively. Figure 6 shows the RMSE graph of LOI. ART performs the worst among the three methods by giving the highest RMSE values whereass (ART + TV)_NLM_ generates the lowest RMSE values. Although RMSE has been in use as a metric for reconstruction accuracy, it has been shown that RMSE may not always been the most appropriate metric for performance comparisons [23,24]. In [25], it was shown that images with the same mean squared error (MSE) values can actually look very different in terms of image quality. Thus a new metric, Structure SIMilarity (SSIM) was introduced [25]. The main characteristic feature of SSIM is to offer a metric which has a closer match with the human vision system. MATLAB code for SSIM can be downloaded from [26]. Figure 7 shows the SSIM value change as the number of iteration increases. ART shows the worst SSIM value performance when it is compared with the other two reconstruction methods. The proposed method gives a slightly better performance than ART + TV.

In image processing literature, the RMSE is mostly converted to a value of peak signal to noise ratio (PSNR). However the PSNR value is useful if images with different dynamic ranges are being compared, otherwise it shows a similar result of what RMSE information provides [23]. For this study the following formulation was used to calculate the SNR between the reconstructed image and the reference image:

(10) $$\mathrm{SNR}=10\log\left(\frac{\sqrt{\sum_{j,k}^{J,K}{\left|X_{{}_{\mathrm{jk}}}^{\mathrm{rec}}\right|}^{2}}}{\sqrt{\sum_{j,k}^{J,K}{\left|X_{\mathrm{jk}}-X_{{}_{\mathrm{jk}}}^{\mathrm{rec}}\right|}^{2}}}\right)$$

**Figure 8:**
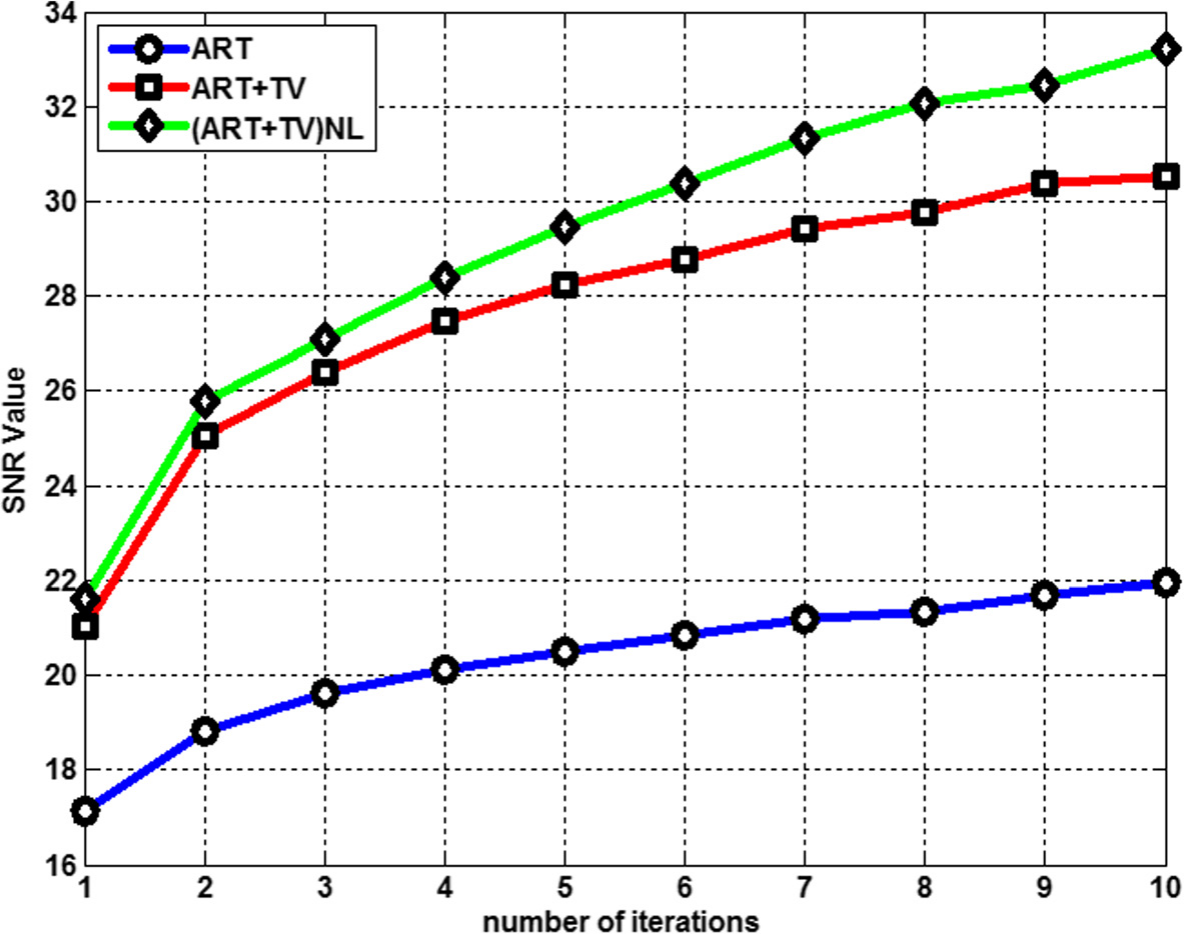
SNR graph of the reconstruction methods.

The formula above uses ratio of the Frobenius norm of the *X*^*rec*^ and the difference between *X* and *X*^*rec*^ in logarithmic form. Figure 8 shows the change of SNR for the three reconstructed images along the number of iterations. The (ART + TV)_NLM_ method gives the highest SNR value. Table 1 gives an overall performance analysis on quantitative results for the LOI at the 10^th^ iteration for ART, ART + TV and (ART + TV)_NLM_. It is very clear from the Table 1 that the best results were obtained for all performance assessment metrics by using the (ART + TV)_NLM_.

**Table 1:** Simulation results

**Numerical results for the LOI at the 10th iteration**			
	ART	ART + TV	(ART + TV)_NLM_
RMSE	0.063	0.027	0.020
SSIM	0.753	0.951	0.960
SNR(dB)	21.95	30.54	33.20

## Discussion and conclusion

Both ART + TV and (ART + TV)_NLM_ methods contain parameters which tune their impact on reconstruction and appropriate selection of parameters has been proven to give better results, though this study does not focus on selecting the optimal values. There have been studies specifically focusing on optimization of parameters for both TV and NLM filtering. Automatically tuning the parameters is beyond the scope of this study. The regularization parameter *λ* which tunes the inclusion of TV was fixed to an experience-based value. In NLM filtering search window size is often limited to decrease the computational time while patch size is chosen smaller than search window to generate a global filtering. The appropriate selection of these parameters has been studied and several approaches have been suggested according to the size of the image to be reconstructed. However the main problem in NLM filtering is the filtering parameter *h* which regulates the smoothness of the reconstruction. The filtering parameter is highly dependent on the image noise, but in this study the dominant noise in the image is not the noise caused by X-ray scattering or system noise but the out-of-focus slice blur. Thus an empirical-based fixed *h* value was used. Optimization of the filtering parameter in tomosynthesis imaging will be considered in our future work.

In this paper, an iterative algorithm based on combining TV minimization and NLM filtering has been applied to the tomosynthesis imaging system. TV minimization step was applied in 3D form while NLM was applied layer by layer in 2D form to cover the entire 3D image. Both methods have the ability to reduce the background noise and each method has also specific abilities as TV preserves the edges while NLM enhances the fine details in the image. The aim in the proposed algorithm was to reduce the out-of-focus slice blur which is the most dominant imaging artifact in tomosynthesis system. The numerical results were conducted to compare the performances of ART, ART + TV, (ART + TV)_NLM_ by using a 3D phantom to simulate the overlapping tissue problem in tomosynthesis imaging. The introduced algorithm, (ART + TV)_NLM_, showed better results than two other reconstruction methods both qualitatively and quantitatively by increasing image quality and by giving smaller RMSE values and higher SSIM and SNR values in the reconstructed images.

## Abbreviations

CS: Compressed sensing; TV: Total variation; ART: Algebraic reconstruction technique; ART + TV: Algebraic reconstruction technique with total variation; NLM: Non-local means; 2D: 2 Dimensional; 2D: 2 Dimensional; 3D: 3 Dimensional; SSIM: Structure similarity; RMSE: Root mean squared error; MSE: Mean squared error; LOI: Layer of interest; FBP: Filtered back projection; MRI: Magnetic resonance imaging; SNR: Signal to noise ratio; FT: Fourier transform; EM: Expectation maximization; POCS: Projection onto convex sets; SART: Simultaneous algebraic reconstruction technique; CT: Computer Tomography; PICCS: Prior image constrained compressed sensing; NLTV: Non-local total variation; SW: Search window; (ART + TV)_NLM_: Introduced method.

## Competing interests

The authors declare that they have no competing interests.

## Authors’ contributions

ME carried out the reconstruction simulations, performed analysis of the simulation results and drafted the manuscript. IY conceived of the study, participated in the design of the study, and helped in drafting the manuscript. MK participated in the design of phantom and system, and helped in drafting the manuscript. AA participated in the coordination and helped in drafting the manuscript. All authors read and approved the final manuscript.
